# Bridging Consciousness and Cognition in Memory and Perception: Evidence for Both State and Strength Processes

**DOI:** 10.1371/journal.pone.0030231

**Published:** 2012-01-17

**Authors:** Mariam Aly, Andrew P. Yonelinas

**Affiliations:** 1 Department of Psychology, University of California, Davis, Davis, California, United States of America; 2 Center for Mind and Brain, University of California, Davis, Davis, California, United States of America; University Of Cambridge, United Kingdom

## Abstract

Subjective experience indicates that mental states are discrete, in the sense that memories and perceptions readily come to mind in some cases, but are entirely unavailable to awareness in others. However, a long history of psychophysical research has indicated that the discrete nature of mental states is largely epiphenomenal and that mental processes vary continuously in strength. We used a novel combination of behavioral methodologies to examine the processes underlying perception of complex images: (1) analysis of receiver operating characteristics (ROCs), (2) a modification of the change-detection flicker paradigm, and (3) subjective reports of conscious experience. These methods yielded converging results showing that perceptual judgments reflect the combined, yet functionally independent, contributions of two processes available to conscious experience: a state process of *conscious perception* and a strength process of *knowing*; processes that correspond to *recollection* and *familiarity* in long-term memory. In addition, insights from the perception experiments led to the discovery of a new recollection phenomenon in a long-term memory change detection paradigm. The apparent incompatibility between subjective experience and theories of cognition can be understood within a unified state-strength framework that links consciousness to cognition across the domains of perception and memory.

## Introduction

Many of life's experiences are associated with qualitatively distinct mental states. When trying to remember our past, we can consciously recollect qualitative details about some events, and yet have little or no recollection for other important events [1]–[4]. When inspecting two very similar photographs, we may be acutely aware of a difference between the two, yet in other cases, fail to notice even pronounced differences [5]–[7]. These examples suggest that some conscious experiences are discrete, and either occur or fail to occur. Yet, a dominant view of cognition is that the appearance of discrete mental states is an epiphenomenon, and cognition in reality varies in a completely continuous manner, such that some memories are simply stronger than others, or some perceptual differences just more noticeable than others ([8]–[12], but see [13]–[14]).

Recent advances in memory research have suggested that pure strength theories may be insufficient, which has led to the development of theories that incorporate both state and strength processes [3]–[4]; [ 15]–[17]. One such theory [3] proposes that in addition to memories that vary continuously in strength, some memories are associated with a discrete mental state. Thus, a given memory judgment can be based on continuously graded information about an event's *familiarity*; alternatively, it can be based on the ability to *recollect* qualitative information about a prior event such as when or where an item was studied, a process that will either occur or fail to occur. The specific information that is recollected or the strength of that information can vary, but most critically, for some items recollection completely fails.

**Figure 1:**
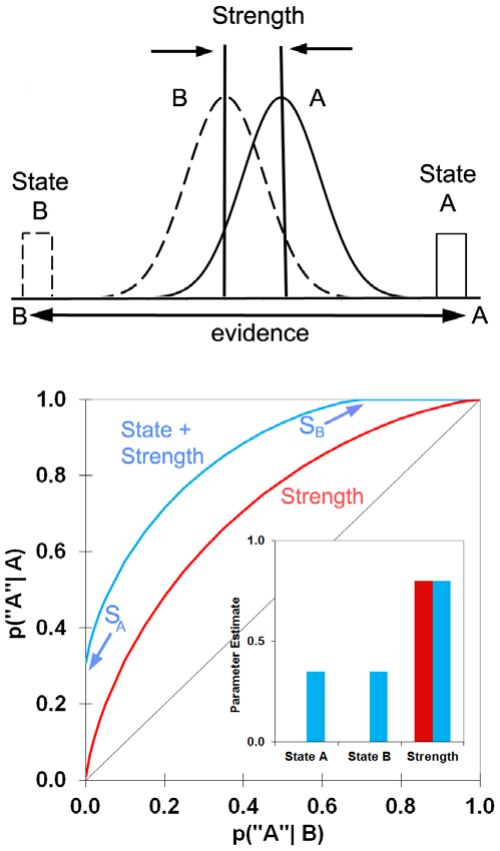
Illustration of state-strength theory. Probability density functions (top) for evidence that stimuli come from class A or B (e.g., old or new items in a test of memory; pairs of same or different images in a test of perception). Classic strength theory postulates continuously-varying evidence distributions for items in different classes (normal distributions for A and B). In contrast, state-strength theory proposes that in addition to continuously-varying distributions, items may elicit discrete mental states (uniform distributions for A and B). Predicted receiver operating characteristics (ROCs) are shown on the bottom. ROCs plot the proportion of correct and incorrect responses across different levels of evidence strength. Strength theory predicts curvilinear ROCs with intercepts at (0,0) and (1,1). However, if some items are associated with a discrete mental state, the ROC intercepts will be shifted so that the left y-intercept occurs at a point corresponding to State A and the upper x-intercept occurs at a point corresponding to State B. The resulting ROC reflects a combination of state and strength processes. Parameter estimates in the inset show estimates of state and strength for these hypothetical ROCs. State and strength processes are, respectively, recollection and familiarity in memory, and perceiving and knowing in perception.

Here, we expand on this approach to propose a general theory of cognition, in which state and strength processes combine independently to support conscious experience in perception and memory (Fig. 1). Our claim is that pure strength theories of cognition provide an insufficient account of perception and memory. We describe a series of experiments which show that although there are conditions under which strength theories work quite well, a full account of perception and memory must include both state and strength processes. In perception, a state process of *perceiving*, similar to recollection (or *remembering* [2]) in memory, provides qualitative, high-resolution information to conscious awareness, but it can often fail entirely. An independent strength process of *knowing*, similar to familiarity (or *knowing* [2]) in memory, provides a quantitative, low-resolution global matching signal. Thus, memory judgments can be based on the recollection of qualitative information about a prior event or on assessments of familiarity, and, similarly, perceptual judgments can be based on the perception of detailed sensory information or on an overall match signal.

In the current work, we first explore the conditions under which strength theories are successful, and other conditions in which both state and strength processes are needed to account for perception. We then examine whether state and strength processes are functionally distinct by testing whether they can be doubly dissociated, whether they exhibit distinct temporal onsets and whether they are associated with different kinds of subjective experiences and sensory information. Finally, we use insights gleaned from state-strength theory in perception to test novel predictions about long-term memory.

## Results

### Investigating when perceiving and knowing contribute to perception

Support for strength theories of perception has come from studies that were designed so that stimuli are ‘just noticeable’ [13], typically by using simple stimuli that are rapidly presented and often immediately masked. One major advantage of this approach is that it affords tight experimental control which greatly simplifies the interpretation of the results. A potential disadvantage of this approach, however, is that the results from these studies may not generalize to real-world perception of more complex materials. Most importantly, we suggest that these data-limited conditions might reduce the contribution of conscious perception [18], and thus they may tell us only half of the story, providing insights about knowing but little insights about perceiving. We therefore tested the prediction that under standard data-limited conditions, perceptual discriminations would be consistent with the dominant strength theory, but that perception of more complex, realistic images would additionally involve a state of conscious perception.

**Figure 2:**
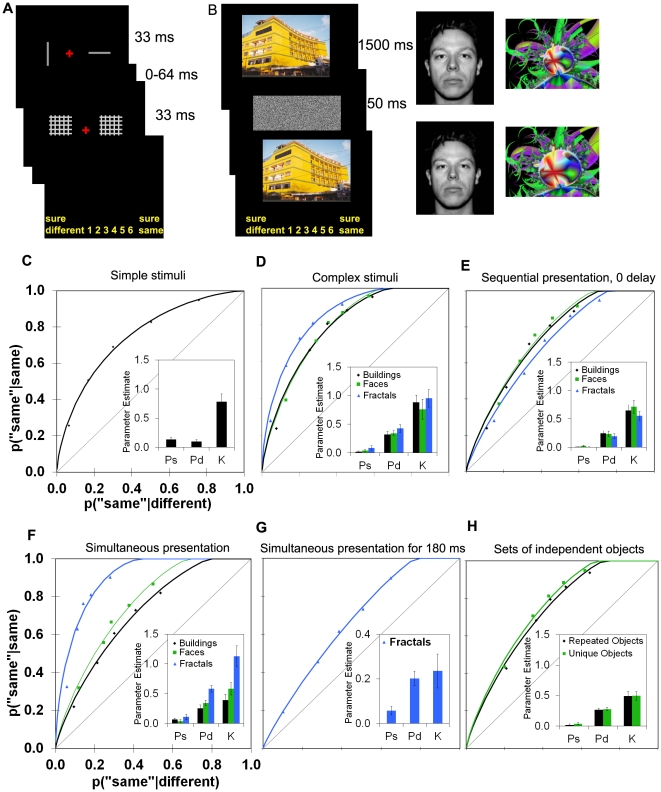
Perception of simple and complex visual images. (**A–B**) Trial procedures and materials for the first series of experiments. (**C–H**) ROCs and estimates of Perceiving Same (Ps, the y-intercept), Perceiving Different (Pd, one minus the upper x-intercept) and Knowing (K, the degree of curvilinearity, measured as *d′)*. Average parameter estimates from individuals' ROCs are in the insets; error bars show the standard error of the mean. Perception of simple stimuli (**C**; trial procedure shown in A) could be accounted for by the strength process of knowing, while perception of complex stimuli such as buildings, faces and fractals (**D**; trial procedure shown in B) was based on knowing as well as a state of perceiving differences. Subsequent experiments indicated that the results with complex stimuli generalized to different presentation conditions and stimuli, including (**E**) sequentially presented images without an intervening mask, (**F**) simultaneously presented images, (**G**) simultaneously presented images at a duration too brief for eye movements to occur, and (**H**) arrays of six objects that were either trial-unique or repeated in different combinations over trials.

To test these ideas, the first experiment examined perceptual discriminations for simple stimuli made under standard psychophysical test conditions [12]–[13] (Fig. 2A; Movie S1). Individuals were shown pairs of rapidly-presented lines and made same/different confidence judgments. The confidence judgments were used to plot receiver operating characteristics (ROCs [19]; Fig. 1) and estimate the contributions of state and strength processes by using curve-fitting algorithms [3], [17]. The ROCs were curvilinear and fit virtually perfectly by pure strength theory [11], [20]–[21] (Fig. 2C). Model fits to the average ROCs verified that judgments were based on a strength process, *knowing*, with essentially no evidence for states of conscious perception [Perceiving sameness (Ps) = 0, Perceiving difference (Pd) = 0, Knowing (K) = 0.97]. Parameter estimates based on individuals' ROCs (Fig. 2C, inset) supported the same conclusions.

To investigate perceptual discrimination of realistic images, the next experiment utilized buildings, faces, and fractals (Fig. 2B; Movie S2). The images were either identical or differed in that one image was slightly expanded or contracted relative to the other. On every trial, individuals were presented with an image for 1500 ms, which was then masked and followed by the second image for a same/different confidence judgment. The ROCs for these perceptual judgments (Fig. 2D) were curvilinear, indicating the contribution of knowing; but most critically, the ROCs now intercepted the top x-axis, showing the contribution of a state process in which individuals were able to perceive when there was a difference between the images. Model fits to the average ROCs verified that judgments were based on perceiving differences (Pd = 0.31, 0.36, and 0.32, for buildings, faces, and fractals, respectively) and on assessments of knowing (K = 0.76, 0.68, and 1.11 for buildings, faces, and fractals, respectively). In addition, the y-intercept was effectively zero, indicating that the state of perceiving did not support identification of sameness (Ps = 0 for all conditions). Parameter estimates based on individuals' ROCs (Fig. 2D, inset) led to the same conclusions.

The conditions in which perceiving and knowing jointly contribute to performance were examined across four additional experiments, which indicated that the pattern of results was robust. In the first of these experiments (Movie S3), the first image was presented very briefly (i.e. 300 ms) and the second image was presented immediately afterward (i.e. a 0 delay, with no intervening mask). The second image was shifted slightly to remove transient motion cues that would otherwise appear on ‘different’ but not ‘same’ trials. Here, too, both perceiving and knowing contributed to performance (Fig. 2E). The same pattern of results held when the two images were presented simultaneously for 1500 ms (Movie S4 and Fig. 2F) and when they were presented simultaneously for 180 ms, a duration that is too brief for voluntary saccades to be made (Fig. 2G). Finally, we used arrays of six objects, one of which could potentially change. In one condition, the objects were trial-unique, while in the other condition, a set of twenty objects was repeated in different combinations of six on each trial (Movie S5 and Fig. 2H). Again, in both of these conditions, perceiving and knowing contributed to performance.

Thus, across a range of different stimuli and presentation conditions, perceiving and knowing contributed to same/different judgments of complex images. Perceiving supported the identification of difference but not sameness, indicating that the perception of a difference is highly diagnostic that images are different, while the perception of similarities between two complex images is not diagnostic that they are identical.

These findings are consistent with the claim that perception of realistic images relies on the contribution of two separate processes. But it is not clear from these data whether perceiving and knowing make independent contributions to performance, or if they are reducible to high- and low-strength perceptions, respectively, on a single underlying strength continuum. If these processes are in fact independent, it should be possible to find a manipulation that affects them in opposite directions.

### Functional independence of perceiving and knowing

The predictions for the next experiment were motivated by theories of memory, which propose that the state process of recollection is associated with access to qualitative details about a prior event, while the strength process of familiarity reflects an assessment of global match between a current event and information in memory [4]. If this extends to perception, the state of perceiving should be associated with access to qualitative details, whereas knowing should be associated with assessments of global match.

**Figure 3:**
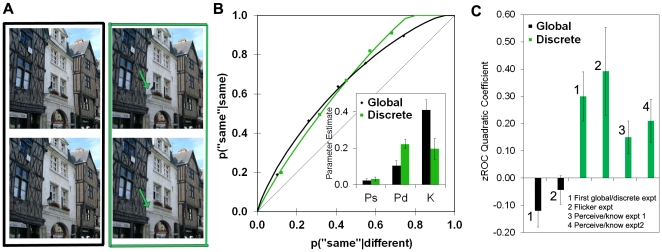
Dissociating perceiving and knowing. (**A**) Examples of global (left) and discrete (right) changes. Buildings were expanded or contracted slightly in the global change condition. A feature was added or removed in the discrete change condition (arrows were not presented in the experiment). The trial procedure was the same as in Fig. 2B. (**B**) ROCs and parameter estimates revealed a crossover dissociation; Pd was significantly greater for discrete compared to global changes [*t*(36) = 3.15, *p* = .003], while K was significantly greater for global compared to discrete changes [*t*(36) = 2.68, *p* = .01]. Ps did not differ, *t*<1. (**C**) Average quadratic coefficients of ROCs plotted in z-space; error bars show the standard error of the mean. Global change zROCs did not differ significantly from linearity [left global bar, *M*_quadratic_ = −0.12, *SE* = 0.06, *t*(18) = 1.84, *p* = .08; right global bar, *M*_quadratic_ = −0.04, *SE* = 0.05, *t*<1, *ns*], whereas discrete change zROCs were U-shaped, i.e. had significant positive quadratic components [from left to right, *M*_quadratic_ = 0.30, *SE* = 0.09, *t*(17) = 3.19, *p* = .005; *M*_quadratic_ = 0.39, *SE* = 0.16, *t*(5) = 2.43, *p* = .05; *M*_quadratic_ = 0.15, *SE* = 0.06, *t*(21) = 2.48, *p* = .022; *M*_quadratic_ = 0.21, *SE* = 0.08, *t*(18) = 2.57, *p* = .019;], ruling against a UVSD strength theory.

We therefore contrasted conditions that varied the degree of global match between images. Each image was altered to form a pair of images that differed in a global manner and a pair of images that differed in a discrete manner. Different individuals took part in the *global change* condition and the *discrete change* condition. The global change condition utilized the types of changes from the prior experiments in which an image was contracted slightly to form one version and expanded slightly to form the other. In contrast, in the discrete change condition, a single feature was added or removed to form the other version of the image (Fig. 3A). In the discrete change condition, the overall perceptual match between ‘different’ stimuli is quite high, so a knowing signal based on global match should not be very useful for detecting differences. In contrast, if individuals perceive the specific, qualitative detail that differs between the two images, the images can be identified as different. In the global change condition on the other hand, an overall global match signal will be useful since the images are the same or different over much of their extent.

The aim was to examine the differential contributions of perceiving and knowing under conditions in which the discrete and global discriminations were equally difficult, in the sense that overall performance is matched for these different types of discriminations. Under these conditions, perceiving should be higher for discrete compared to global changes, and knowing should be higher for global compared to discrete changes. It is important to note that these conditions are not expected to be process-pure; that is, both discrete and global change conditions should be associated with contributions from both perceiving and knowing. The critical prediction is that the *relative* contributions of these processes can be doubly dissociated, so that perceiving makes a relatively larger contribution in the discrete change condition, and knowing makes a relatively larger contribution in the global change condition.

In line with predictions, this manipulation led to crossover dissociations in the ROCs and in the parameter estimates (Fig. 3B). Discrete changes were associated with a larger contribution of perceiving than global changes, and global changes were associated with higher levels of knowing than discrete changes. This double dissociation cannot be attributed simply to differences in strength, because difficulty was matched; rather, it indicates that there must be two functionally independent components underlying performance.

A strength account of perceptual judgments, however, might still be possible if ‘different’ trials are associated with greater variability in matching strength than ‘same’ trials. We assessed this *unequal variance signal detection theory* (UVSD; [16]) by examining ROCs plotted in z-space (zROCs). The UVSD theory predicts linear zROCs, while state-strength theory predicts U-shaped zROCs when performance is driven by a state process. In line with the latter, zROCs in the global change condition did not deviate significantly from linearity, but zROCs in the discrete change condition exhibited a significant U-shape (Fig.3C, first experiment). Moreover, in all of the subsequent experiments that included global and discrete changes (see below), discrete change zROCs were consistently U-shaped, whereas the global change zROCs were not (Fig. 3C). The U-shaped zROCs verify the *a priori* prediction of state-strength theory and are inconsistent with the strength-only UVSD theory.

### Measuring perceiving and knowing across time

If perceiving is a discrete mental state, it should be possible to find situations in which perceiving has a relatively sudden temporal onset. Individuals may therefore show abrupt transitions from a state of not perceiving into a state of perceiving. In the current tasks, where the detection of any difference is diagnostic that images are different, perceiving should have a sudden onset when a critical difference between two images is identified. In contrast, if knowing is a strength process based on global match, then the strength of this signal should increase gradually as additional information is accumulated over time.

In the next experiment, we contrasted a condition associated primarily with perceiving (i.e. discrete changes) to a condition associated with both perceiving and knowing (i.e. global changes). The former should show mostly ‘step function’ transitions for ‘different’ trials, in which individuals show relatively abrupt transitions from a state of being unsure to a state of high-confidence correct information (i.e. perceiving). In contrast, the latter condition should show both step function transitions and more gradual transitions from low to intermediate and then to high confidence (i.e. knowing). For both conditions, ‘same’ trials should show primarily gradual transitions, consistent with the previous findings that perceiving does not contribute significantly to the identification of sameness.

To test these predictions, we used a modification of the flicker paradigm [6] which allowed confidence judgments to be tracked over repeated exposures to a given pair of images (Movie S6). Each trial consisted of ten repetitions of a pair of either ‘same’ or ‘different’ images. The differences were discrete or global, for different individuals. Following each of the ten presentations of each pair, individuals made a same/different confidence response on a 9-point scale.

**Figure 4:**
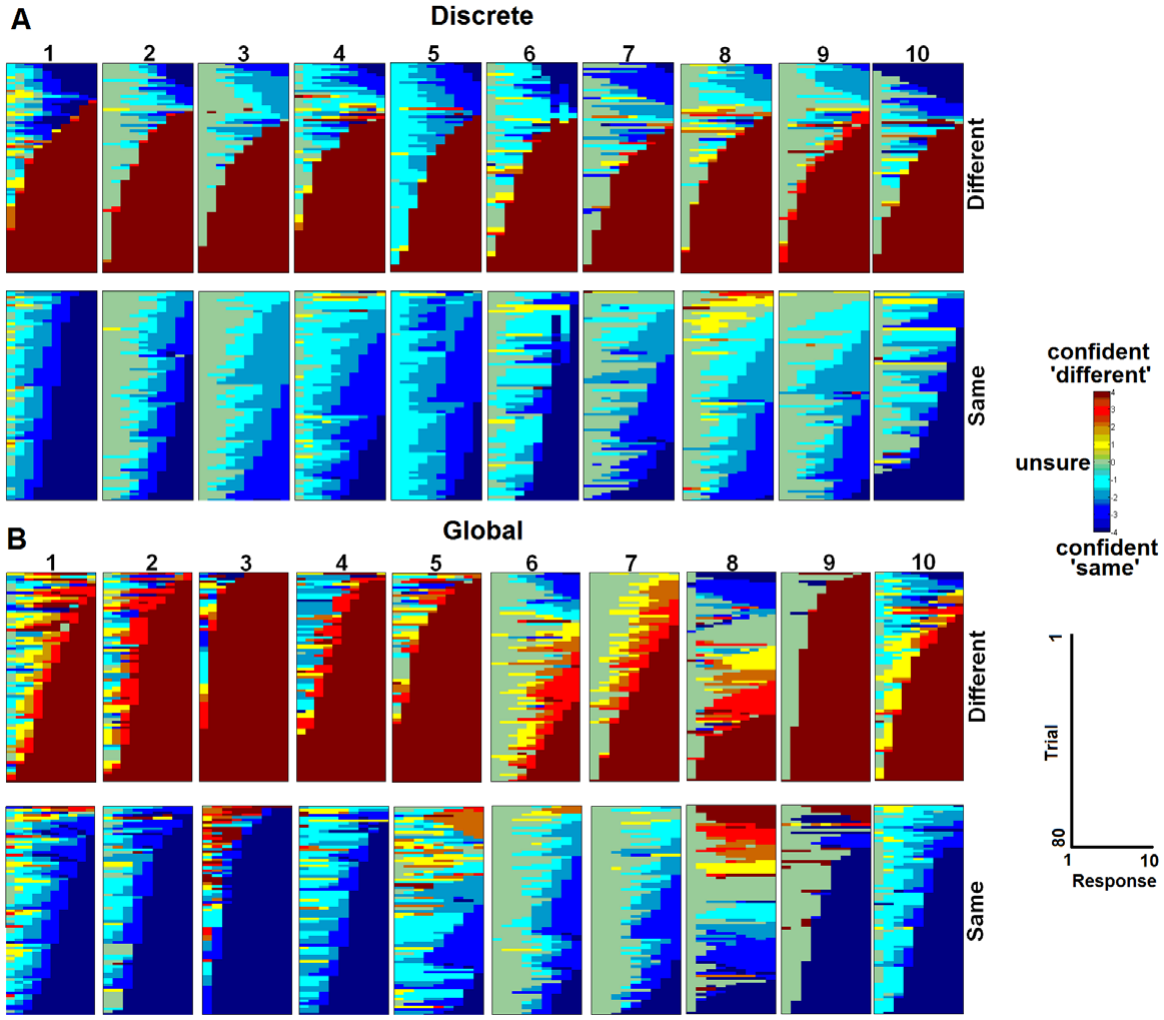
Tracking perceiving and knowing over time. Same/different confidence responses across repetitions for each trial in the flicker paradigm. Data are shown for the ten individuals (numbers on top of each column of blocks) tested in the discrete change condition (**A**) and the ten individuals tested in the global change condition (**B**). ‘Different’ trials are the top row of blocks for each of the discrete and global change conditions; ‘same’ trials are the bottom row of blocks for each condition. Trials are sorted so that the fastest learning trials appear on the bottom of each block. In each block, every row is a trial, and the x-axis represents responses 1 through 10. Unsure responses are green, hotter colors indicate more confident ‘different’ responses; cooler colors indicate more confident ‘same’ responses. In the discrete change condition, the correct identification of differences showed an abrupt, step function transition to high confidence responses. In contrast, in the global change condition, the correct identification of differences showed step function transitions on some trials and more gradual transitions on other trials. The identification of sameness gradually transitioned from low to intermediate to high confidence for both conditions.

Fig. 4A–B shows confidence ratings across the image repetitions. When the two images in a pair were different (top row of blocks in Fig.4A and B), a majority of trials exhibited a discrete step function, in which responses abruptly transitioned to the highest-confidence ‘different’ response in a transition of two or more steps in confidence [*M(SE)* was 67(3)% and 56(8)% of ‘different’ trials for discrete and global changes, respectively]. In contrast, when the two images were the same (bottom row of blocks in Fig.4A and B), step functions were significantly less likely [11(7)%, *t*(9) = 7.46, *p*<.001 for discrete changes, and 29(8)%, *t*(9) = 3,45, *p* = .007 for global changes]. Instead, the majority of ‘same’ trials that obtained a high confidence ‘same’ response [47(12)% and 65(10)% of all ‘same’ trials for discrete and global changes, respectively], were associated with gradual transitions from low to intermediate and then to high confidence [36(10)% and 36(8)% of all ‘same’ trials for discrete and global changes, respectively], and this did not differ for discrete and global changes [*t*(18) = .02, *ns*]. Moreover, gradual identification of differences was more likely to occur for global compared to discrete changes [25(7)% and 6(3)% of ‘different’ trials for global and discrete changes, respectively, *t*(18) = 2.60, *p* = .018]. Thus, perceiving differences can be supported by a process that onsets abruptly, whereas detection of similarity is based on a process that builds more gradually. In addition, when changes in a scene are global rather than discrete, there is gradual learning of differences as well as similarities.

**Figure 5:**
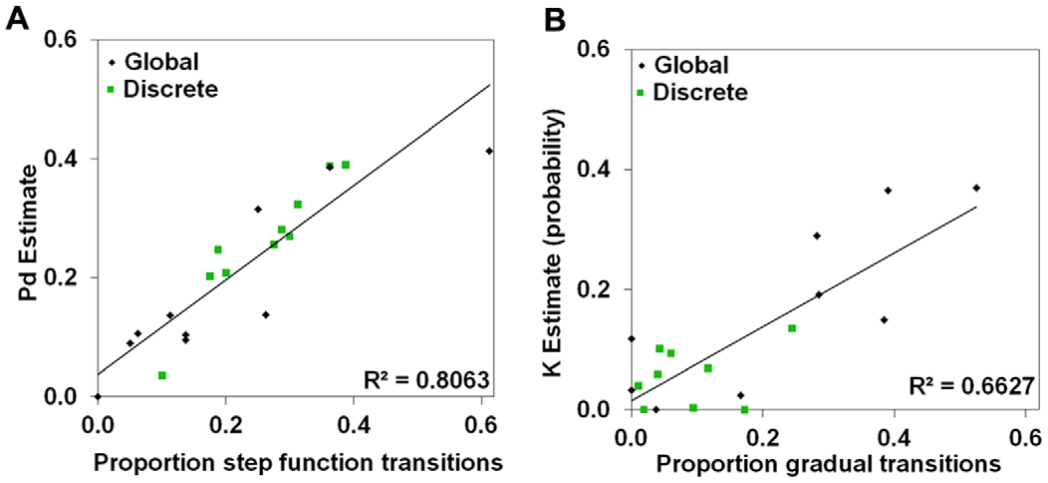
Relating step functions and gradual transitions to perceiving and knowing. (**A**) ROC estimates of perceiving were highly correlated with the proportion of step function transitions, *r* = .898, *p*<.001. (**B**) ROC estimates of knowing were highly correlated with the proportion of gradual transitions (right), *r* = .814, *p*<.001.

To assess whether step function and gradual transition trials reflected perceiving and knowing, respectively, we examined the relationship between these trial types and estimates of perceiving and knowing derived from an ROC analysis. The proportion of ‘different’ trials in which high-confidence responses appeared suddenly was correlated with ROC estimates of perceiving difference (Fig. 5A), whereas the proportion of gradual transition trials was correlated with ROC estimates of knowing (Fig. 5B). Importantly, the converse correlations were not statistically significant; step function transitions were not significantly correlated with ROC estimates of knowing, and gradual transitions were not significantly correlated with estimates of perceiving, both *p*s>.2. These findings suggest that perceiving is a discrete mental state that onsets suddenly, while knowing is a continuously graded mental process that builds gradually over time.

**Figure 6:**
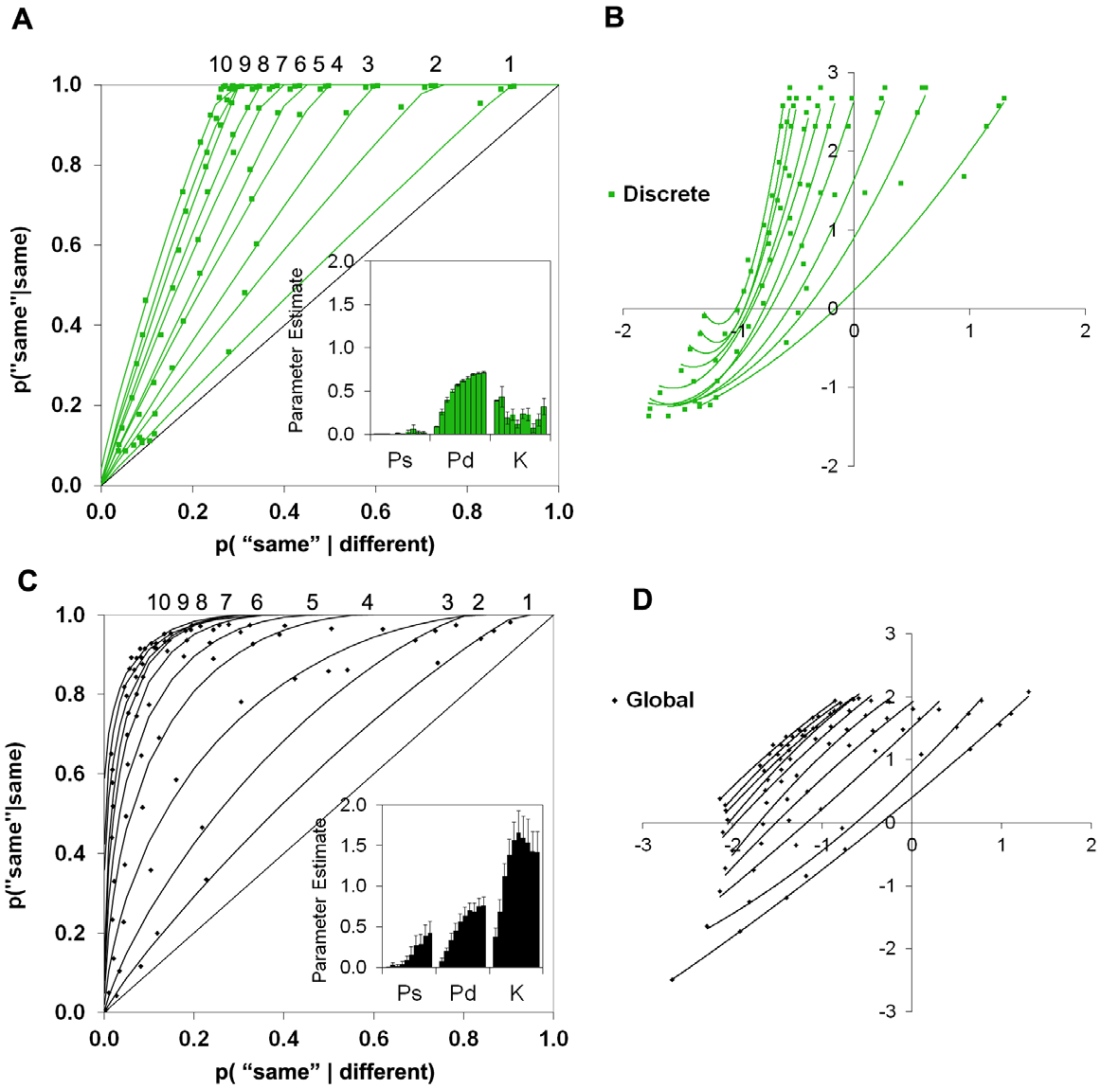
ROCs and zROCs over time. ROCs (left) and zROCs (right) were plotted for the discrete (top row) and global (bottom row) change conditions for each of the ten responses in the flicker paradigm. Average estimates of perceiving and knowing are in the insets of the ROCs; error bars show the standard error of the mean. The discrete change ROCs (**A**) were consistently linear, and the resulting zROCs (**B**) were U-shaped. The global change ROCs (**C**) were curvilinear with an upper x-intercept, and the zROCs (**D**) did not deviate from linearity.

The sudden onset of perceiving compared to knowing suggests that as more features of the images are sampled, individuals tend to suddenly notice differences between paired images. However, once a difference is noticed, could a simple strength process account for performance? To assess this, we examined ROCs for each repetition within a trial. Inconsistent with a strength account, discrete changes produced relatively linear ROCs at all levels of performance (Fig. 6A). Moreover, the global change ROCs were found to be consistently curvilinear and had an intercept at the top x-axis (Fig. 6C), indicative of performance supported by both perceiving and knowing across levels of performance. Parameter estimates confirmed that the discrete change condition was supported more by perceiving than knowing, while the global change condition was associated with both perceiving and knowing (Fig. 6A and C, insets). Finally, replicating and extending the findings from the first global/discrete experiment (see Fig. 3C), the zROCs for the discrete change condition were consistently U-shaped (Fig. 6B) while the global change zROCs were not (Fig. 6D).

### Conscious awareness of perceiving and knowing

In the previous experiments, perceiving and knowing were inferred from the ROCs and learning functions. In order to assess whether perceiving and knowing reflect distinct subjective experiences accessible to conscious awareness, we developed a *perceive/know* paradigm, inspired by the remember/know paradigm in studies of memory [2]. In the remember/know paradigm, individuals introspect on their subjective experiences while making recognition judgments. ‘Remember’ responses are given when individuals recollect specific details about where or when an event was encountered before, while ‘know’ responses are given when individuals experience something as familiar but are not able to retrieve details about the episode in which it was initially seen. Similarly, in the current study, *perceive* was defined as being consciously aware of specific, qualitative details that serve as a basis for responding, while *know* was defined as ‘just knowing’ that images were either different or the same, but not being able to provide any specific details about why.

**Figure 7:**
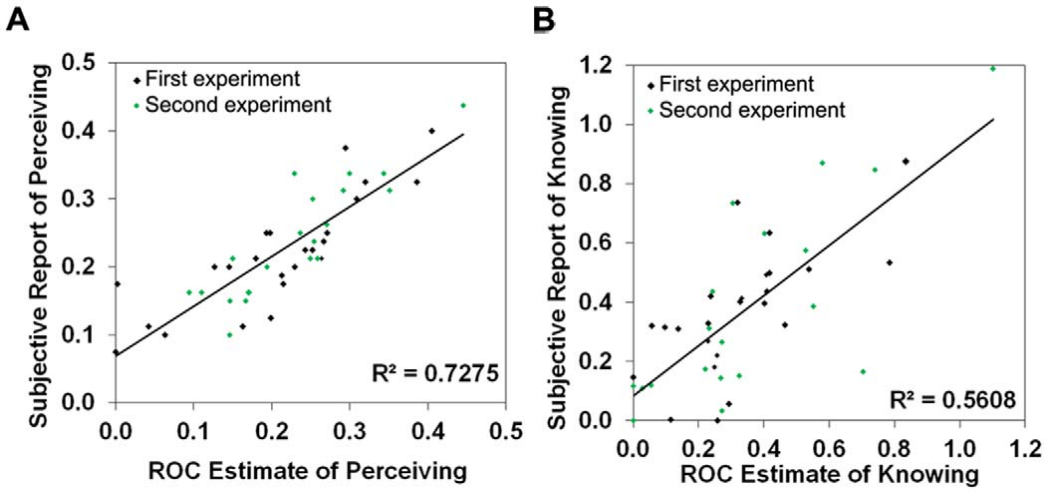
Subjective availability of perceiving and knowing. Correlations between ROC estimates and subjective reports of perceiving (**A**) and knowing (**B**). There was a positive correlation between ROC estimates and subjective reports of perceiving differences [Pd, *r* = .87, *p*<.001; *r* = .82, *p*<.001 for the first and second experiments, respectively] and of knowing [*r* = .67, *p*<.001; *r* = .79, *p*<.001 for the first and second experiments, respectively].

In the current experiment, we examined the detection of discrete changes, since these types of changes are the most amenable to the qualitative, verbal descriptions necessary to justify ‘perceive’ responses. On every trial, individuals gave confidence responses to pairs of buildings, and then introspected on their subjective perceptual experiences to provide a perceive/know report. Confidence responses were used to plot ROCs to yield estimates of perceiving and knowing, and these estimates were compared to the subjective reports of perceiving and knowing. If perceiving and knowing, as measured in the ROCs, are psychologically real in the sense that they are available to subjective experience, there should be a direct relationship between the ROC estimates and subjective reports of conscious experience. As predicted, we found a strong positive correlation between the ROC estimates and estimates from subjective reports (Fig. 7A–B, first experiment), showing that perceiving and knowing are associated with phenomenologically distinct experiences.

To determine whether subjective reports of perceiving are associated with conscious access to veridical information [22], another group of individuals completed the perceive/know experiment, but additionally reported the aspect of each image that had been altered when a ‘different’ response was given. Replicating the previous experiment, there was a close correspondence between the ROC estimates and subjective estimates of perceiving and of knowing (Fig.7A–B, second experiment). Importantly, the proportion of trials in which individuals correctly identified the specific detail that had changed was significantly correlated with ROC estimates of perceiving, *r* = 0.57, *p*<.01, but was not significantly correlated with estimates of knowing, *r* = 0.24, ns. Moreover, for the ‘different’ trials leading to a ‘perceive different’ response (proportion = 0.29), in almost every case (0.26), individuals correctly identified the specific detail that had changed. In contrast, for the ‘different’ trials leading to a ‘know different’ response (0.25), individuals were just as likely to provide incorrect details (0.06) as correct details (0.06), and were twice as likely to provide no detail at all (0.13). Thus, when conscious perception occurs, individuals accurately report the specific detail that was altered, whereas when responses are based on knowing, this ability is absent or reduced.

In the memory literature, it has been argued that subjective reports of familiarity may sometimes be associated with recollection of qualitative information, and, conversely, that subjective reports of recollection may be given even when events are only familiar [23], [24]. Might this ‘contamination’ criticism apply here, for reports of perceiving and knowing? We would not argue that subjective reports of perceiving and knowing are process pure, but several aspects of the current results indicate that reports of perceiving were relatively uncontaminated by knowing. For example, memory research has shown that when careful instructions are given to individuals [23], [25], such contamination is minimized. In the current studies, we were careful to provide individuals with strict instructions; namely, that ‘perceive’ responses should only be given if a verbal description that justifies the response can be provided. In addition, while ROC and subjective report estimates of perceiving (and knowing) correlated highly, there was no significant correlation between ROC estimates of perceiving and subjective reports of knowing, or ROC estimates of knowing and subjective reports of perceiving (all *p*s>.16). If reports of perceiving and/or knowing were contaminated, one would predict to see significant correlations between reports of perceiving and ROC estimates of knowing, and likewise with reports of knowing and ROC estimates of perceiving. Finally, the results from the detail reports suggest that subjective reports of perceiving and knowing are associated with access to qualitatively different kinds of information.

### Using insights from perception to reveal recollection of items not previously studied: A long-term memory change detection paradigm

The current state-strength theory was motivated by a theory of long-term memory [3], but the perception results are quite different from those seen in studies of memory in an important way. That is, in studies of item recognition memory, observed ROCs are invariably found to be curvilinear with a y-intercept [16], rather than an upper x-intercept, as observed in the perception studies reported here. These results suggest that in memory, the state process of recollection supports the identification of oldness (i.e. that a test item is the same as a studied item), whereas in perception, the state process of perceiving supports identification of newness (i.e. that items are different from one another). Why would the state processes in memory and perception, and the resulting ROCs, differ in this way?

We propose that the reason is that the detection of similarities and differences tend to play opposite roles in memory and perception. That is, in perceptual tasks, noticing even a small change between two images is sufficient to make a definitive ‘different’ response. On the other hand, noticing similarities between two very similar, complex images is not definitive evidence that they are identical, as there may be differences that were simply not noticed. Thus, in these perceptual tasks, one expects the state of perceiving to support the detection of difference (i.e. an upper x-intercept) rather than the detection of sameness. In recognition memory tasks, on the other hand, the test list typically contains a mixture of multiple studied items and multiple items that are completely new to the experiment. Under these conditions, recollecting that a test item is the same as a studied item is quite useful in making a recognition judgment, and this will produce a positive y-intercept. In contrast, failing to recollect a test item is not particularly diagnostic; recollection may have failed not because the item is new, but because the item was not adequately encoded at study or it was forgotten over the delay. The only way one could recollect that a test item was not in the study list is if one could remember every one of the studied items and by a process of elimination reject the test item as new. Thus, in recognition memory tasks, one expects the state of recollection to support the detection of oldness (i.e. a y-intercept) rather than the detection of newness.

**Figure 8:**
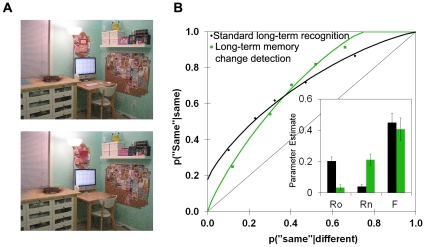
Generalizing from perception to long-term memory. (**A**) Example of a scene from the long-term memory change detection paradigm. In the example shown here, the bottom image is missing a few items that are present in the top image, including the keyboard, the mouse, and the wall outlets. (**B**) ROCs in long-term memory change detection and in a performance-matched standard recognition memory procedure. Change detection compared to standard recognition led to an increase in recollection of new items [Rn; *t*(52) = 5.37, *p*<.001] and a decrease in the recollection of old items [Ro; *t*(52) = 4.67, *p*<.001] whereas estimates of familiarity did not differ [F; *t*<1]. Average parameter estimates are in the inset in (B); error bars show the standard error of the mean.

If this account is correct, it should be possible to observe a state process in memory that supports recollection of newness, and eliminate the use of recollecting oldness, if we could find a set of conditions under which memory for newness becomes diagnostic. To this end, we chose to try to make the recollection of newness diagnostic in memory in the same way that the perception of difference was useful in the current perceptual tests. That is, rather than having the test list made up of distinct study and test items, we designed an experiment in which the test list was made up of studied items and very similar items that had been modified in some subtle way, similar to the discrete change condition in the earlier perception experiments (see Fig. 8A). We predicted that under these conditions, recollection that something had changed between study and test would be diagnostic, which would lead to an upper x-intercept reflecting the contribution of recollection of newness. Conversely, because the images are complex, recollection of oldness would no longer be diagnostic that the test image is identical to the studied image in every way, because there may have been differences that were not noticed. The y-intercept should now approach zero, indicating that recollection of oldness is no longer useful. As far as we know, no study of recognition memory has ever shown this pattern of results [16]; however, the contributions of recollection and familiarity in a long-term memory change detection paradigm have never been examined.

To test these ideas, we first examined standard long-term item recognition for the buildings used in the previous perception studies. Individuals studied a series of buildings, and at test, were shown a list of old, studied buildings randomly intermixed with entirely new, unstudied buildings. Confidence judgments at test were used to plot ROCs. In line with prior long-term memory research [16], we found that the ROCs were asymmetrical with a positive y-intercept, indicating recollection of old, but not new, items (Fig. 8B, standard long-term recognition ROC).

A separate group of individuals studied a list of complex scenes and were tested on their memory for scenes that were either presented exactly as studied, or were changed so that elements were either added or removed (Fig. 8A and Movie S7). Here, identification of any difference is diagnostic that the scene is different (i.e. new), but identification of similarities is not diagnostic that the image is exactly the same (i.e. old). In contrast to standard item recognition, the resulting memory ROCs were now shaped like perception ROCs, indicating that individuals recollected new items as new, but did not recollect old items as old (Fig. 8B, long-term memory change detection ROC). Importantly, overall performance in the two recognition tests was matched, such that the ROCs crossed over. Thus the differences in shape cannot be attributed to differences in overall memory strength. The discovery of this new memory phenomenon (long-term change detection, or recollection of newness) reveals the utility of adopting a unified theoretical model (i.e. state-strength theory) of both perception and memory.

## Discussion

Together, these experiments provide evidence that there is no incompatibility between the discrete nature of subjective experience and the continuous phenomena that are often examined in studies of memory and perception. The general theoretical framework proposed here suggests that both state and strength processes make independent contributions to perception and memory. We found that perceptual judgments are supported by a discrete state of perceiving that becomes available relatively abruptly, and a sense of knowing that increases gradually over time. As with studies of recollection and familiarity in long-term memory [4], perceiving and knowing were functionally independent and available to conscious awareness. Moreover, as with recollection, when conscious perception occurred, it was associated with accurate access to qualitative details.

Whether this theoretical approach proves useful in other cognitive domains remains to be tested, but there is reason to be optimistic that it might be quite general. For example, in studies of working memory, state models assuming a fixed memory capacity [26] and strength models assuming a large capacity [27]–[28] have been proposed, and each has found considerable empirical support. A hybrid model that incorporates both state and strength processes might allow for an integration of these two approaches [29]. In the visual attention literature, a controversial proposal has been that detecting changes in visual scenes may be supported by a process of sensing a change in the absence of conscious experience, or by conscious visual experience of qualitative information about the change ([30], but see [31]). These two processes may depend on pre-attentive visual processes that allow access to low-level object information, and attentional mechanisms that form coherent conscious percepts, respectively [6], [32]–[33]. Determining how these attentional processes are related to the processes of knowing and perceiving awaits future studies.

The current findings suggest that a single theoretical framework – state-strength theory – is able to account well for the results from both perception and memory. We believe that finding such a unifying framework is an important theoretical advance that informs the understanding of memory and of perception. It tells us that at some fundamental level, both memory and perception can be characterized as reflecting a combination of state and strength processes. The ability to take insights from memory and apply them to perception, and then in turn to take insights from perception and apply them to memory tells us that this framework is quite useful. This does not, however, imply that the state and strength processes in perception are identical to those in memory. We expect that they are quite different, and future work that aims to examine how they do differ will be informative.

That perceiving and knowing make independent contributions to perception has important implications for researchers investigating the neural basis of complex perceptual discriminations. For example, memory research has shown that recollection and familiarity have distinct neural substrates [see 34 for review]. It seems reasonable that perceiving and knowing may likewise have distinct neural underpinnings. This possibility remains to be explored in patient and neuroimaging work. We suspect that qualitative differences in how the brain supports perceptual judgments will be overlooked if one does not take into account the distinction between perceiving and knowing.

### Comparison to alternative theoretical frameworks

The current theoretical approach differs in important ways from other accounts of perception and memory. As discussed earlier, a dominant approach has been to treat perception and memory as reflecting the assessment of a single underlying strength continuum [8]–[12]. In the current studies, we show that this dominant approach is inadequate when one examines perception of complex, realistic images. This does not imply that this approach is not useful in some situations; indeed, under standard psychophysical test conditions [13] this theory does extremely well, suggesting that in data-limited situations, performance can be based largely on a simple strength process. What our results do show is that this approach is insufficient when one moves beyond the realm of rapidly-presented, masked, simple stimuli. Perception of more realistic images depends on a combination of state and strength processes. Thus, there are important differences between perception of real-world images and perception in the kinds of data-limited conditions traditionally used in laboratory psychophysics experiments. A pure strength theory cannot explain the double dissociations observed in the global/discrete experiments, the distinct patterns of confidence transitions over time, the different conscious experiences available to awareness, or the different types of information that are accessible when perceiving and knowing occur. Thus, these findings show that pure strength theories are not sufficient when rich, ecologically valid stimuli are the objects of perception.

State-strength theory is also distinct from several models that have focused on the distinction between conscious and unconscious processes [18], [35]–[36]. Although the current approach recognizes as fundamental the distinction between conscious perception/recollection on the one hand and knowing/familiarity on the other, it treats the conscious/unconscious distinction as more of a continuum than a true dichotomy [18]. That is, perceiving and knowing are associated with different kinds of consciousness, rather than the presence or absence of conscious experience. Perceiving carries with it consciousness of *what* has changed, while knowing may only be associated with consciousness that there has been a change. This distinction is related to that between phenomenal and access consciousness [37]. Knowing is associated with phenomenal consciousness (namely, an experience of perceptual similarity or difference) but not access consciousness - specific details are not available for verbal report. Perceiving, on the other hand, is associated with both phenomenal and access consciousness.

Just as perceiving and knowing are associated with different kinds of consciousness, recollection and familiarity in memory are related to different types of conscious experiences. Recollection is accompanied by *autonoetic*, or ‘self-knowing’ consciousness, while familiarity is accompanied by *noetic*, or ‘knowing’ consciousness [2]. Here, too, the distinction between state and strength processes does not map directly on to conscious and unconscious processes, respectively, but rather different kinds of consciousness.

### Future directions

The studies reported here suggest a number of important questions for future research. For example, how do the state and strength processes identified here relate to processes underlying unconscious perception/memory? The current experiments focused on separating state and strength processes as they contribute to performance on conscious (or explicit) perception and memory tasks. In studies of memory, familiarity is found to be related to some forms of implicit memory (i.e. memory without awareness). For example, both familiarity and conceptual implicit memory are found to be influenced by similar experimental variables [4] and they are both critically dependent on the perirhinal cortex [38]. Thus, in memory at least, it appears that familiarity and implicit forms of memory may share similar underlying processes. Whether this is true for knowing and unconscious perception is unknown, but one might expect that the same neurocognitive processes supporting knowing judgments might also support forms of unconscious perception, possibly differing only in that knowing responses might be stronger than manifestations of unconscious perception. Future studies examining this possibility will be important. A related question worth examining is whether there may be unconscious perceptual processes that exhibit state-based characteristics, or whether unconscious processes might necessarily be strength based.

Finally, state-strength theory does not indicate how these two types of processes arise and so computational models that specify how state and strength signals can be derived will also be critical. One possibility is that the determination of sameness or difference depends on local sequential sampling and comparison of paired stimuli. Perceiving difference might occur when the system samples several identical features before sampling a large difference between paired stimuli; this would result in a sudden transition from information about ‘sameness’ to information about ‘difference’, and could be associated with access to specific local details that differ between two stimuli. On the other hand, knowing difference or sameness might occur when local sequential sampling returns a small ‘different’ or ‘same’ signal on each sample, and this signal accumulates gradually as more and more features are sampled. This would result in gradual transitions in which increasing evidence for ‘difference’ or ‘sameness’ is accrued over time. This is only one of many potential mechanisms that might underlie perceiving and knowing. The important point is that the current data show evidence for two qualitatively different end states - one that onsets suddenly and is associated with access to detailed information, and another which grows gradually over time and is associated with access to global matching information. A successful computational model must be able to produce these two distinct temporal onset patterns that are associated with access to different kinds of information.

### Conclusion

We propose that a general distinction in types of information available to consciousness is useful for understanding phenomena in both perception and memory. One type of information is a result of relatively local access to high-resolution, qualitative details, and is associated with a state of perceiving or of recollection. Another type of information is characterized by relatively global or low-resolution match signals, and is associated with strengths of knowing or of familiarity. We speculate that this single theoretical framework is useful in relating memory and perception because both cognitive functions are dependent on these common principles. Our findings suggest that the critical question in cognition might not be whether a given experience is ‘perception’ or ‘memory’, is ‘conscious’ or ‘unconscious’, but whether the mental experience is characterized by a discrete mental state or a process that varies continuously in strength.

## Methods

### Ethics Statement

Treatment of all participants was in accordance with the ethical standards of the American Psychological Association. Written informed consent was obtained after the nature and possible consequences of the study were explained. The studies were approved by the University of California, Davis Institutional Review Board.

### Experiment 1 - Perceptual judgments of simple stimuli

#### Participants

Twenty two undergraduate students from the University of California, Davis participated in the experiment for credit in an introductory psychology course. All participants had normal or corrected-to-normal vision. Different sets of participants from the same participant pool served in all the subsequent experiments. Three participants were excluded for performing at chance levels and one participant was excluded for not using the confidence scale as instructed.

#### Materials, Design, and Procedure

The experiment consisted of two blocks of 100 trials each. The stimuli were two dark gray lines, each either vertical or horizontal, presented to the left and right of a central fixation cross. Vertical and horizontal lines appeared equally often in the left and right locations. Half of the trials were ‘same’ trials, in which both lines were the same orientation, and half were ‘different’ trials, in which one line of each orientation was presented. Same and different trials were presented in a random order. All trial types (i.e. two vertical, two horizontal, vertical on left and horizontal on right, horizontal on left and vertical on right) occurred equally often.

Each trial began with a 1500 ms centrally-presented fixation cross. Two lines then appeared for 33 ms to the left and right of fixation. Following an inter-stimulus interval of 0, 16, 33, 48, or 64 ms, two dynamic grid masks were presented for 33 ms over the locations of the target lines. A confidence scale then appeared, and individuals made a self-paced same/different judgment using a 1 (‘sure different’) to 6 (‘sure same’) confidence scale (Fig. 2A and Movie S1). The scale was displayed on the screen while individuals made the confidence response, and each point on the scale was labeled (1 = sure different, 2 = maybe different, 3 = guess different, 4 = guess same, 5 = maybe same, 6 = sure same). The same confidence scale was used in all the subsequent ROC studies unless otherwise noted. Additionally, in this and all subsequent studies, the scale was carefully explained to participants at the beginning of the experiment, and they were encouraged to use all six keys over the course of each block.

The ISI was determined before the experiment using a titration procedure. The titration phase was used because pilot studies showed that for any one study duration, some participants performed perfectly while others were at chance. Five levels of ISI were tested. Level 0 was 33 ms, and there were two levels below and two levels above in steps of 16 ms (i.e. 0, 16, 33, 48, and 64 ms ISIs). Each participant started with a 20-trial titration block at Level 0. Trial procedure was identical to the experimental procedure described above, except that participants received feedback on accuracy and cumulative accuracy on a trial-by-trial basis. At the end of the 20 trials, if accuracy was between 65% and 75%, the participant repeated another 20-trial titration block with the same ISI. If the participant fell above the 65%–75% range, the titration block was repeated with the ISI one step below. If the participant fell below the 65%–75% range, the titration phase was repeated with the ISI one step above. Titration blocks were repeated, in increasing or decreasing steps, until the participant reached 65%–75% accuracy for two successive 20-trial blocks. Once this occurred, the participant was started on the experimental phase with an ISI one step below the one at which they reached criterion (note that participants who reached criterion at 0 ms necessarily had a 0 ms ISI during the experiment). This procedure ensured that performance was not at ceiling or at floor for the experimental phase.

#### Data Analysis

Confidence ratings were used to plot receiver operating characteristics (Fig. 1). The leftmost point corresponds to the probability of a hit (y-axis) and false alarm (x-axis) for the most confident ‘same’ response, and subsequent points are the cumulative probabilities for the hits and false alarms with responses of decreasing confidence. In this and all subsequent ROC studies, the ROCs were fit to state-strength theory using a nonlinear regression method that minimizes the sum of squared errors (SSE) between the predicted function and observed data points [3], [17]. Maximum likelihood estimation on the aggregate data led to comparable results as the SSE method in all experiments, thus only the SSE results are reported.

### Experiment 2A – Perceptual judgments of buildings, faces and fractals

#### Participants

Eighteen participants took part in the experiment. Four participants were excluded for using only two of the six response keys.

#### Materials

Three types of materials were used: buildings, faces, and fractals. One hundred and sixty of each stimulus type served as experimental stimuli, and six of each stimulus type served as practice stimuli. Colored photographs of buildings were obtained from Internet searches. Face photographs were grayscale, frontal images of males and females with neutral facial expressions, which were cropped at the neck. The male faces were found from Internet searches and from databases available courtesy of Michael J. Tarr (Center for the Neural Basis of Cognition and Department of Psychology, Carnegie Mellon University, http://www.tarrlab.org/). The female faces were obtained from the University of Texas at Dallas Center for Vital Longevity face database [39]. The fractals were made using the Tiera-Zon Fractal Generator freeware program.

For each stimulus, two altered versions were created in Adobe Photoshop. The first version was expanded outward slightly using the ‘spherize’ option. The second version was contracted inward slightly using the ‘pinch’ option. Pilot studies were conducted to find levels of distortion that led to equivalent levels of performance (measured in *d′*) for the buildings, faces, and fractals.

#### Design and Procedure

The experiment was divided into three blocks (buildings, faces, and fractals), with order of the blocks counterbalanced across participants. Each block consisted of one hundred and sixty trials. Eighty trials were ‘same’ trials in which identical stimuli were presented (i.e. both pinched versions or both spherized versions; these trial types occurred equally often). Eighty trials were ‘different’ trials in which the two altered versions of a stimulus were presented (i.e. the pinched version followed by the spherized version, or vice versa; these trial types occurred equally often). Pinched and spherized stimuli occurred equally frequently as the first and second stimuli. Two stimuli lists were created so that each stimulus was tested on both ‘same’ and ‘different’ trials across participants. Same and different trials were presented in a random order.

Participants were told that they would be presented with pairs of very similar images, and had to judge if the two images were the same or different. On each trial, participants viewed a ‘get ready’ screen for 1500 ms. This was followed by a building, face, or fractal for 1500 ms, then a dynamic noise mask for 50 ms. The dynamic mask consisted of three different noise masks presented for 17 ms, 17 ms, and 16 ms. The corresponding identical (on ‘same’ trials) or alternate (on ‘different’ trials) version of the stimulus was then presented, and participants gave a response using a 1 (‘sure different’) to 6 (‘sure same’) scale to indicate their confidence that the two stimuli were the same or different (see Fig. 2B and Movie S2). Responses were self-paced and the second stimulus and the response scale stayed on the screen until a response was made.

Before the experiment, participants viewed sample buildings, faces, and fractals on the computer. One pair of ‘same’ stimuli and one pair of ‘different’ stimuli for each of the material types were used as examples of the types of stimuli in the experiment. Participants were encouraged to scroll through the images and observe the differences between pairs of images, so that they knew what types of changes to expect in the experiment. Participants also completed a short (four trial) practice block, using the trial procedure specified above, before each block of the experiment. All participants completed all three blocks, with a short break in between the blocks.

### Experiment 2B – Sequential presentation with 0 delay

#### Participants

Eighteen participants took part in the experiment, but one participant was excluded for using only two of the six response keys.

#### Materials, Design, and Procedure

The materials, design, and procedure were identical to Experiment 2A with the following exceptions. The first stimulus was presented for 300 ms instead of 1500 ms, and the masks were removed so that there was no delay between the first stimulus and the second stimulus (see Movie S3). In order to prevent participants from using transient motion cues to detect changed stimuli, the second stimulus was shifted slightly to the right of the first stimulus, so that there was motion on both ‘same’ and ‘different’ trials.

### Experiment 2C – Simultaneous Presentation

#### Participants

Nineteen participants took part in the experiment, but three participants were excluded for using only two of the six response keys.

#### Materials, Design, and Procedure

The materials, design, and procedure were identical to Experiment 2A with the following exceptions. The two stimuli were presented side by side, to the left and right of fixation, for 1500 ms (see Movie S4), after which the confidence scale appeared on the screen. Responding was self-paced and the scale stayed on the screen until individuals made a response, but the stimuli were not on the screen for the response.

### Experiment 2D – Simultaneous Presentation for 180 ms

#### Participants

Eighteen participants took part in the experiment, but three participants were excluded for using only two of the six response keys.

#### Materials, Design, and Procedure

The materials, design, and procedure were identical to Experiment 2C with the following exceptions. Only the fractals were used, and the exposure duration was reduced to 180 ms. The distortions to the fractals were increased so that performance would not be at floor. The distortions were made using the ‘pinch’ and ‘spherize’ options in Photoshop, as in the previous experiments.

### Experiment 2E – Sets of independent objects

#### Participants

Twenty nine participants took part in the experiment, but two participants were excluded for using only two of the six response keys.

#### Materials

Eight hundred photographs of objects were obtained from the BOSS database [40], from databases available courtesy of Michael J. Tarr (Center for the Neural Basis of Cognition and Department of Psychology, Carnegie Mellon University, http://www.tarrlab.org/), and from Internet searches. All objects were on a white background and were converted to grayscale and resized to 120 by 120 pixels using Adobe Photoshop. Twenty of the objects were used in the ‘repeated’ condition, and the remaining seven hundred and eighty were used in the ‘unique’ condition. An additional sixteen objects were used for a five-trial practice block. The practice images ([41], also courtesy of Michael J. Tarr, see above) were converted to grayscale line drawings using the ‘photocopy’ command in Adobe Photoshop. The practice images were chosen to be distinct from the experimental images.

#### Design and Procedure

The experiment was divided into two blocks, with a short practice phase at the beginning of the experiment. The stimuli in the ‘repeated’ block were twenty objects that were used repeatedly over the block in different combinations of six on each trial. In the ‘unique’ block, the objects were unique on every trial. Block order was counterbalanced across participants. Each block consisted of sixty ‘same’ trials and sixty ‘different’ trials, presented in a random order.

Participants were told that they would be presented with arrays of objects, and they had to judge if the two arrays presented on a trial were the same or different. They were told that the arrays would either be exactly the same, or one object will be different. On each trial, a fixation cross appeared in the center of the screen, with six objects arranged around it (see Movie S5). This study array was presented for 1000 ms, and then a dynamic noise mask was presented for 50 ms at each of the six locations, followed by a test array of six objects. On ‘same’ trials, the test array was identical to the study array. On ‘different’ trials, one of the six objects was replaced with a different object. Each of the six locations was equally likely to change over the course of the experiment. In the ‘repeated’ block, each stimulus was equally likely to appear in each of the six locations and equally likely to appear as the new stimulus on ‘different’ trials.

Participants used a 1 (‘sure different’) to 6 (‘sure same’) scale to indicate their confidence that the two arrays were the same or different. Responses were self-paced and the test array and response scale stayed on the screen until a response was made. Participants were encouraged to use all six keys over the course of the block. An inter-trial interval of 1000 ms elapsed before the next study array appeared. At the end of the block, participants had a short break before completing the next block.

### Experiment 3A – Global vs. discrete changes

#### Participants

Thirty eight participants took part in the experiment, half in the ‘global change’ condition, and the other half in the ‘discrete change’ condition.

#### Materials, Design, and Procedure

The one hundred and sixty original (unaltered) building images from Experiment 2A were used to create different changed versions for the ‘global change’ and ‘discrete change’ conditions. Thus, the same original images were used for the global and discrete change conditions but different changes were made to the stimuli for the two conditions.

The global changes were the ‘pinch’ and ‘spherize’ distortions from Adobe Photoshop, which were used in the earlier studies. These options alter an image so that it is different over much of its extent. For the discrete changes, using Adobe Photoshop, a change was made to each building so that an element of the scene was either added or removed (Fig. 3A). Since pilot studies indicated that the discrete change condition was more difficult than the global change conditions used in previous studies, the global changes were more subtle in this experiment compared to what had been used in the other studies.

Two stimuli lists were created so that each stimulus was tested on both ‘same’ and ‘different’ trials across participants. Half of the trials were ‘same’ and the other half were ‘different’, and these trials were randomly presented. For the discrete change condition, the unaltered original image was presented first on half of the trials, and the changed version was presented first on the remaining half. Half of the ‘same’ trials involved presentation of two original images and the other half involved presentation of the two altered images. Half of the ‘different’ trials involved presentation of the original image first and the altered version second, and the other half presented the altered image first and the original image second. The same stimulus counterbalancing was used for the global change condition except that the ‘spherized’ and ‘pinched’ buildings were used in place of the unaltered originals and discrete change buildings.

The trial procedure was identical to Experiment 2A (Fig. 2B and Movie S2). On each trial, participants viewed a ‘get ready’ screen for 1500 ms. This was followed by a building for 1500 ms, which was then masked for 50 ms. The corresponding identical (on ‘same’ trials) or alternate (on ‘different’ trials) version of the stimulus was then presented, and participants gave a response using a 1 (‘sure different’) to 6 (‘sure same’) scale to indicate their confidence that the two stimuli were the same or different. Responses were self-paced and the second stimulus and the response scale stayed on the screen until a response was made.

Before the experiment, participants viewed four pairs of sample buildings on the computer. The same original building images were used to create different changed versions for the global and discrete change conditions. For the global change condition, a ‘pinched’ and ‘spherized’ version of each building were viewed. For the discrete change condition, the original building and an altered version of the building (i.e. something was added or removed) were viewed. Participants were encouraged to scroll through the images and observe the differences between pairs of images, so that they knew what types of changes to expect in the experiment. Participants also completed a short (four trial) practice block before the experiment, using the trial procedure specified above.

#### Data Analysis

Since different groups of individuals were tested in the global and discrete change conditions, statistical tests were independent-samples *t*-tests, with *n* = 19 in each of two groups. The alpha level was 0.017, two-tailed. This alpha level was chosen based on the Bonferroni correction (i.e. *p* = .05/3 comparisons of interest, for Ps, Pd, and K). Exact *p*-values are reported in the paper.

zROCs were obtained by converting the probability ROC points to z-scores, and plotting the ROCs in z-space. The z-ROC quadratic analyses were one-sample *t*-tests, used to determine if the quadratic coefficients were significantly different from zero. The alpha level was 0.05, two-tailed, for each test.

In this and all subsequent experiments, the data were checked for normality using the Shapiro-Wilk normality test. Since normality is difficult to accurately assess with small samples, in all cases we also analyzed the data with the non-parametric Mann-Whitney *U*-test. In no case were the results of the statistical tests altered, so we report parametric tests in the paper.

### Experiment 3B - Assessing state and strength processes using a flicker paradigm

#### Participants

Twenty participants took part in the experiment, half in the ‘global change’ condition, and the other half in the ‘discrete change’ condition.

#### Materials, Design, and Procedure

The materials were identical to Experiment 3A. Participants took part in either the discrete change condition or the global change condition. Two stimuli lists per condition were again used, divided equally among the participants. As in Experiment 3A, participants viewed sample buildings before beginning the experiment. Each participant completed 160 trials (80 different and 80 same), with 10 responses per trial.

The trial procedure was a modification of the flicker paradigm used in change-detection studies [6]. A trial consisted of 10 repetitions of the following sequence: an image for 150 ms, a black screen for 80 ms, the corresponding ‘same’ or ‘different’ image for 150 ms, and, finally, a 9-point confidence scale for a self-paced same/different judgment (Movie S6). Following the 10 repetitions, the next trial began, using the same procedure.

For the confidence judgment, participants were told to use the four fingers on their left hand for ‘different’ responses, the four fingers on their right hand for ‘same’ responses, and the spacebar for ‘I don't know.’ The keys *a*, *s*, *d*, and *f* were used for ‘sure different’, ‘maybe different’, ‘slight chance difference’, and ‘guess different’, respectively. The keys *l*, *k*, *j*, and *h* were used for ‘sure same’, ‘maybe same’, ‘slight chance same’, and ‘guess same’, respectively. The scale and the corresponding keys were displayed on the screen while individuals made the confidence response (i.e. ‘a’ was labeled as ‘sure different’ and so on).

#### Data Analysis

Confidence ratings were re-coded so that 1, 2, 3, and 4 indicated increasing levels of confidence that a pair was different, and −1, −2, −3, and −4 indicated increasing levels of confidence that a pair was the same. ‘I don't know’ responses were coded as 0.

To examine how correct high-confidence responses appeared over time, each trial was classified as either a ‘step function’ trial or a ‘gradual transition’ trial depending on how the transition to the highest-confidence correct response arose. Trials that did not end on the highest-confidence correct response were not used in this analysis. Confidence transitions of one step were defined as ‘gradual’ (i.e. from ‘3’ to ‘4’ for ‘different’ trials and from ‘−3’ to ‘−4’ for ‘same trials). Confidence transitions of two steps or greater were defined as ‘step functions’. The proportion of step function and gradual transition ‘different’ and ‘same’ trials were calculated for each participant by dividing the number of those trials by the total number of trials in that condition. Planned comparisons were carried out on the proportions (see main text). To ensure that the results did not depend on using raw proportions, the data were also examined in two other ways. First, the occurrence of gradual transitions was calculated conditional on a step function transition not occurring. Second, the raw proportions were corrected for false alarms by subtracting the proportion of incorrect responses (e.g. subtracting the proportion of incorrect gradual transitions on ‘same’ trials from the proportion of correct gradual transitions on ‘different’ trials). The results of the statistical tests were not affected by which of these methods was used.

Since different groups of individuals were tested in the global and discrete change conditions, transition analyses were conducted using independent-samples *t*-tests when comparing global and discrete conditions (*n* = 10 in each of the two groups), and paired-samples *t*-tests when comparing ‘same’ and ‘different’ trials within the global and discrete conditions. The alpha level for the two between-groups comparisons was 0.025, two-tailed, using the Bonferroni correction for two comparisons. The alpha level for each within-group comparison was 0.05, two-tailed (i.e. one comparison for each group). Exact *p*-values are reported in the paper.

The correlation analysis examined the relationship between step function transitions and perceiving, and between gradual transitions and knowing. The second response in each series of ten repetitions was used because it was the first response that avoided floor effects. Step function and gradual transition trials were defined as above, except that gradual transition trials also included correct ‘different’ responses made with lower confidence (i.e. 1s, 2s, and 3s). Proportions of step function transitions and conditional proportions of gradual transitions (see above) were used in the correlation analyses. False alarm rates were corrected for as described above. Estimates of Pd, Ps, and K were obtained from the ROCs on the second response. K was converted to a probability so as to be on the same scale as the other estimates. To convert the *d′* value to a probability, each individual's false ‘different’ rate (i.e. 1–4 responses on ‘same’ trials) was used to find the hit rate that would yield that *d′* value. The false alarm rate was then subtracted from the hit rate to yield an estimate of K as a probability. Probabilities of Pd and K were then correlated with probabilities of step function and gradual transition trials as described above.

The z-ROC quadratic analyses (Fig. 3C) were one-sample *t*-tests, used to determine if the quadratic coefficients were significantly different from zero. The alpha level was 0.05, two-tailed, for each test.

#### Additional Analyses

Since the proportion of trials showing gradual transitions must decrease as the proportion of trials showing step function transitions increases, a difference in the proportion of gradual transitions between the discrete and global conditions may be observed if they differed in the occurrence of step function transitions. To verify that the difference in gradual transitions is a true difference, we examined the conditional probabilities of gradual transitions, given that step function transitions did not occur. Global changes were still associated with more gradual transitions than discrete changes, and if anything, this difference was magnified in the conditional proportions [14(5)% and 62(11)% of ‘different’ trials for discrete and global changes, respectively, *t*(18) = 3.96, *p*<.001].

Step function transitions in the flicker data analyses were defined arbitrarily as transitions of two steps or greater. In order to precisely quantify step function transitions, we examined the average step size for transitions greater than one step (note: transitions of one step in confidence are the minimum that must be reserved to define ‘gradual transitions’, so we examined the size of all other transitions). Specifically, we examined the jump in confidence points from the response preceding the highest-confidence correct response (i.e. a ‘4’ for ‘different’ trials) to that final high-confidence response. For example, a transition from ‘0’ to ‘4’ would be a step of size four, a transition from ‘−1’ to ‘4’ would be a step of size five, and so on. The average size of step function transitions was *M* = 4.43, *SE* = 0.13 for discrete changes and *M* = 3.76, *SE* = .21 for global changes. This suggests that using transitions of two steps or greater to define ‘step functions’, while arbitrary, would not have led to different results than choosing steps of size three or four. Choosing transitions of step size four may have slightly reduced the number of step function transitions for the global change condition, but this would only magnify the difference between conditions in the predicted direction.

### Experiment 4A - Subjective reports of perceiving and knowing

#### Participants

Twenty seven participants took part in the experiment, but three participants were excluded for chance performance.

#### Materials, Design, and Procedure

The materials were the discrete change buildings materials from Experiment 3. These materials were chosen because the discrete changes were most amenable to the qualitative, verbal descriptions required to justify ‘perceive’ responses.

The perceive/know instructions were explained in detail to the participants. Participants were told to give a *perceive* response if they had an experience of consciously perceiving the images as being either different or exactly the same and were able to tell the experimenter specific details about how the images were either different or the same. They were told to only give this response if they perceived specific details about how the images were either different or the same. For the example shown in Fig. 3A, a justification of a ‘perceive different’ response could be, ‘There used to be a lamp by the windowsill, and then it was gone’.

Participants were instructed to give a *know* response if they just knew that the images were either different or the same, but were not able to provide specific details about why they thought the images were either different or the same. It was emphasized that they could be very confident that the images were different or the same, but if they could not give specific details about how the images were different or the same, they should give a ‘know’ response.

Before the experiment, participants viewed four pairs of sample buildings. The pairs consisted of a building and an altered version of that building (i.e. something was added or removed). Participants were encouraged to scroll through the images and observe the differences between pairs of images, so that they knew what types of changes to expect in the experiment. Participants then completed a short (four trial) practice block. After the practice block, each participant was asked to justify the perceive/know response that they had given on the last practice trial. Acceptable answers for ‘perceive’ judgments were those that included descriptions of specific details about how the images were the same or different. Acceptable answers for ‘know’ judgments were those in which a participant expressed that the images seemed either the same or different but could not provide specific details about why. If a participant did not fully understand the instructions, as evidenced by their responses, the instructions were repeated to ensure that the distinction between perceive and know was understood.

#### Data Analysis

Confidence data were used to plot ROCs as in previous experiments. Ps, Pd, and K were estimated from the perceive/know responses based on a modification of the Independence Remember/Know (IRK) procedure in long-term memory research [42]. Ps and Pd were estimated the same way that recollection is estimated from Remember responses:
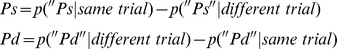
K was estimated in a similar way as familiarity is estimated, that is, conditional on Ps and Pd not occurring. The K probability was converted to a *d*′ score using the inverse of the standard normal cumulative distribution (i.e. ‘normsinv’ in the equation below), and subtracting false alarms from hits.
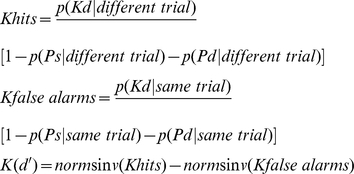



### Experiment 4B – Verifying the basis of subjective reports

#### Participants

Twenty participants took part in the experiment.

#### Materials, Design, and Procedure

The materials, design, and procedure were identical to Experiment 4A, except that participants additionally gave a detail report following each ‘perceive different’ or ‘know different’ response to indicate the basis for their response. For example, a participant may give a ‘perceive different’ response and follow that with a detail report in which they describe a window disappearing from the front of a house. Alternatively, a participant may give a ‘know different’ response and follow that with a report of just ‘knowing’ that the images were different but not being able to provide a specific reason why. Participants gave a verbal response on each trial, which was recorded by the experimenter.

#### Data Analysis

The confidence responses were analyzed as in the previous experiments. Perceive and know estimates from the subjective reports were obtained as in Experiment 4A. For each pair of images, two scorers (M.A. and a research assistant), wrote detailed descriptions of the specific aspect of the image that changed from the original to the altered version of the image. These descriptions were refined until both scorers agreed on the wording.

To score the detail reports, the transcribed report of the participant was compared to the written descriptions. When the report matched the specific detail that had changed, the response was scored as a ‘correct detail’. When the report was of another (unaltered) detail in the image, one that did not match the actual changed detail, the response was scored as an ‘incorrect detail’. Reports in which no specific detail was reported were scored as ‘no detail’. The scorers were always able to reach agreement in situations where there was an initial discrepancy in the scoring.

### Experiment 5A – Standard long-term recognition memory

#### Participants

Thirty six participants took part in the experiment, but two participants were excluded for performing at chance levels.

#### Materials

The materials were the one hundred and sixty buildings used in Experiments 2A–C and one hundred new buildings found from Internet searches. Half of the buildings were altered using the ‘pinch’ option in Adobe Photoshop, and the other half were altered using the ‘spherize’ option, as in the earlier experiments. Two stimuli lists were created so that each stimulus was tested on both ‘same’ and ‘different’ trials across participants. ‘Same’ and ‘different’ trials were presented in a random order. The one hundred new buildings were used as lures in the recognition memory test.

#### Design and Procedure

The first block was identical to the buildings block in Experiment 2A, and served as an incidental encoding phase for a subsequent surprise long-term recognition memory test. One hundred buildings from the same/different task were randomly selected and mixed with one hundred new buildings for a recognition test. Half of the old and half of the new buildings were ‘pinched’, and the remaining half of the old and new buildings were ‘spherized’, so that participants could not use these distortions as a basis for recognition judgments. Participants viewed each building one at a time and rated their confidence that the building had been seen as part of the same/different task, using a 1 (‘sure new’) to 6 (‘sure old’) scale. Each building and the confidence scale stayed on the screen until the participant made a response.

### Experiment 5B – Long-term memory change detection

#### Participants

Twenty one participants took part in the experiment, but one participant was excluded for performing at chance.

#### Materials

One hundred and twenty color photographs of complex indoor and outdoor scenes were collected from Internet searches and an online database ([43], courtesy of Michael J. Tarr, Center for the Neural Basis of Cognition and Department of Psychology, Carnegie Mellon University, http://www.tarrlab.org/). The scenes were selected so that they contained many elements that could potentially change. For each scene, a changed version was created in Adobe Photoshop, where multiple elements of the scene were altered by either adding or removing objects.

#### Design and Procedure

The experiment was divided into twelve study-test blocks. Each block consisted of ten scenes in the study phase and all ten in the test phase. Half of the images at test were identical to the studied image, and half were different. Of the ‘same’ trials, half consisted of the presentation of both original images, and half consisted of the presentation of both altered images. Half of the ‘different’ trials were ‘additions’, in which some elements were added to the scene which were not present at study. The remainder of the ‘different’ trials were ‘deletions’, in which some elements of the studied scene were removed in the test scene. Half of the studied images were the original images, and the other half were the changed images. Half of the tested images were the original images, and the other half were the changed images. This ensured that participants could not identify images as different by looking for cues that the image was edited in Photoshop. Two stimuli lists were created so that each stimulus was tested on both ‘same’ and ‘different’ trials across participants. ‘Same’ and ‘different’ trials were presented in a random order.

For each of the twelve study-test blocks, participants studied ten scenes for seven seconds each, and were instructed to study each scene carefully for a subsequent memory test (see Movie S7). The test consisted of all ten scenes presented in a random order. For each scene, participants responded using a 1 (‘sure different’) to 6 (‘sure same’) scale to indicate if the image was exactly the same as the one they had studied, or was different in any way. The scale and the scene stayed on the screen until a response was made.

Before the experiment, participants viewed three pairs of sample images. The pairs consisted of a scene and an altered version of that scene, wherein some elements had been added or removed. Participants were encouraged to scroll through the scenes and observe the differences between pairs of scenes, so that they knew what types of changes to expect in the experiment.

#### Data Analysis for Experiments 5A–B

Statistical tests were independent-samples *t*-tests, with *n* = 34 in the standard long-term recognition condition and *n* = 20 in the long-term memory change detection condition. The alpha level was 0.017 for two-tailed comparisons, based on the Bonferroni correction (i.e. *p* = .05/3 comparisons of interest, for Ro, Rn, and F).

## Supporting Information

Movie S1 **Trial procedure for Experiment 1.**
(MP4)

Movie S2 **Trial procedure for Experiment 2A.**
(MP4)

Movie S3 **Trial procedure for Experiment 2B (brief sequential presentation and no mask).**
(MP4)

Movie S4 **Trial procedure for Experiment 2C (simultaneous presentation).**
(MP4)

Movie S5 **Trial procedure for Experiment 2E (sets of objects).**
(MP4)

Movie S6 **Trial procedure for Experiment 3B (global change condition in the flicker paradigm).**
(MP4)

Movie S7 **Trial procedure for Experiment 5B (long-term memory change detection).**
(MP4)
